# Quantification of ambient PM_2.5_ concentrations adjacent to informal brick kilns in the Vhembe District using low-cost sensors

**DOI:** 10.1038/s41598-023-49884-7

**Published:** 2023-12-17

**Authors:** Tolulope Elizabeth Aniyikaiye, Stuart J. Piketh, Joshua Nosa Edokpayi

**Affiliations:** 1 Department of Geography and Environmental Science, University of Venda, Private Bag X5050, Thohoyandou, 0950 South Africa; 2 Unit for Environmental Sciences and Management, Climatology Research Group, North-West University, Potchefstroom, 2531 South Africa; 3 Water and Environmental Management Research Group, Department of Geography and Environmental Science, University of Venda, Private Bag X5050, Thohoyandou, 0950 South Africa

**Keywords:** Climate sciences, Environmental chemistry, Lasers, LEDs and light sources

## Abstract

The widespread exposure to ambient PM_2.5_ poses a substantial health risk globally, with a more pronounced impact on low- to medium-income nations. This study investigates the spatiotemporal distribution of PM_2.5_ in the communities hosting informal brickmaking industries in Vhembe District. Utilizing Dylos DC1700, continuous monitoring of PM_2.5_ was conducted at nine stations adjacent to informal brick kilns from March 2021 to February 2022. The study determined the correction factor for PM_2.5_ measurements obtained from the Dylos DC1700 when it was collocated with the GRIMM Environmental Dust Monitor 180. Additionally, the diurnal and seasonal variations across monitoring stations were assessed, and potential PM_2.5_ sources were identified. The study also evaluated the compliance of ambient PM_2.5_ concentrations across the stations with the South African National Ambient Air Quality Standard (NAAQS) limits. Annual PM_2.5_ concentrations for the stations ranged from 22.6 to 36.2 μgm^−3^. Diurnal patterns exhibited peak concentrations in the morning and evening, while seasonal variations showed higher concentrations in winter and lower concentrations in summer and spring. All monitoring stations reported the highest daily exceedance with respect to the daily NAAQS limit in the winter. Major PM_2.5_ sources included domestic biomass combustion, vehicular emissions, industrial emissions, and construction sites. Well-calibrated low-cost sensors could be employed in suburb regions with scarce air quality data. Findings from the study could be used for developing mitigation strategies to reduce health risks associated with PM_2.5_ exposure in the area.

## Introduction

Globally, exposure to ambient fine particulate matter (PM_2.5_) is a major health challenge^1,2^ due to its minute size and potential of penetrating through the human lungs into the circulatory system^3,4^. PM_2.5_ is both a primary pollutant, emitted directly from sources and a secondary pollutant formed from gaseous precursors^5,6^. Ambient PM_2.5_ concentration provides a reliable indication of air quality^7^. According to the World Health Organization^8^, more than 90% of people worldwide live in areas where PM_2.5_ standards are exceeded. PM_2.5_ was reported to be responsible for an estimated 5 million premature deaths and 147 million disability-adjusted life years in 2017^9–11^. It is well established that PM_2.5_ exposure has both acute and chronic health consequences^12–15^_._ Exposure to PM_2.5_ can trigger and/or aggravate a wide range of respiratory system-related disorders, including asthma, lung cancer, silicosis, irritating mucous membrane of the respiratory track, inflammation of the mucous membrane, bronchitis, pulmonary emphysema, lung inflammatory reactions, chronic obstructive pulmonary disease (COPD), and pneumonia^4,16^. Cardiopulmonary diseases such as intravascular thrombin formation, ischemic heart disease, cerebrovascular infection, cardiac dysrhythmias, congestive heart failure, and stroke have also being linked to PM_2.5_ exposure^17–19^. The ability to efficiently identify PM_2.5_ sources and quantify PM_2.5_ concentrations in the ambient environment can facilitate the development of mitigatory measures needed to minimise exposures and the resultant health effects^20^.

Attempts to collect ambient real-time air quality data with high-resolution is often encumbered by the limitations of the air quality instruments^21^. Ambient air quality monitoring is often assessed using a limited number of stationary air quality monitoring stations, basically because the widespread deployment of these regulatory-grade air monitoring sensors is too expensive^1,22–24^. In the developing world, especially in Africa, the use of the conventional methods of air quality monitoring is too sparse to meet the growing challenges of development^25,26^. This toughens assessing the gravity of the air pollution problem, as there is limited or no evidence to show that air quality is a challenge, thus creating a harmful cycle^23,27^. In South Africa, the available air monitoring stations are mostly located in and around major industrial and densely populated urban centers^25,28,29^. Several studies in South Africa have also assessed ambient PM_2.5_ concentrations using a single or network of gravimetric equipment around low-income settlements^30–32^. The Vhembe District of Limpopo, on the other hand, do not receive much preference. Although, particulate matter monitoring occurs in some districts in Limpopo, the Vhembe District currently has no functional ambient air quality monitoring station for PM_2.5_^29^. To generate a more robust PM_2.5_ database with high temporal-spatial resolution, a relatively inexpensive and readily operated real-time measuring apparatus for PM_2.5_ monitoring is essential.

By addressing cost concerns and incorporating portability, the low-cost sensors (LCS) are increasingly gaining global acceptance in their use for air quality assessment^33–36^. The easy operation of LCS promotes public participation^37^, easy accessibility to air quality data and public awareness^38^. Additionally, LCS enable extensive monitoring of air pollution with higher spatiotemporal resolution, thus augmenting data gathered through conventional technology^22,39^. The robust PM_2.5_ database generated using PM LCS, enables policymakers to target interventions at highly polluted regions, ultimately improving air quality^40^. Dylos DC1700 (Dylos Corporation, Riverside, CA) is one of the commonly used LCS for monitoring particulate matter. Dylos being an optical particle counter operates by illuminating a single particle at a time to count the number of particles in defined size bins^41^. The primary challenge with light-scattering instruments is that the instrument response relies upon both the size distribution and the number of particles, instead of the cumulative mass of aerosol. This limitation could be minimised, and reliability enhanced through periodic calibration against a federal reference method^36,42,43^. Dylos is also limited by its detection limit of 5 µm, which makes it impossible to measure ultrafine particles that pose a greater risk to humans^34,44^. Dylos is most conveniently used for indoor air quality monitoring due to the short battery life and its fragility to withstand outdoor environment. The use of Dylos for monitoring indoor air quality has been reported in a number of studies^22,34,45,46^. When used for outdoor air quality monitoring, the study is often conducted in partially enclosed environments, or used in the outdoor environment for relatively short period of time during dry weather^47–49^. To guarantee the sensor’s long-term effectiveness, further investigation on Dylos stability is required^38^.

Brickmaking in many developing nations is acknowledged as a polluting industry characterised by small-scale enterprises that are challenging to monitor and regulate^50–54^. In 2010, the clamp kiln, a commonly used brickmaking technology was enlisted among the activities requiring an Atmospheric Emissions Licensing (AEL) for operation in South Africa^55^. The brickmaking industry is known to be one of the thriving commercial activities in the Vhembe District. However, there is limited knowledge on the impact this industry has on ambient PM_2.5_ in communities close to the brick kilns. This study is born out of the limited database on PM_2.5_ level in ambient air around the brickmaking industry in Vhembe District. The study assessed the ambient PM_2.5_ around the informal clamp kilns, using a network of Dylos DC1700 PM sensor for a period of 1 year. For the first time in Africa, a correction factor for PM_2.5_ readings from Dylos DC1700 PM sensor was generated. The research also examined the extended suitability of Dylos DC1700 for outdoor monitoring of ambient PM_2.5_ concentrations.

The objectives of the study include the following:

- To conduct 1-year continuous monitoring of PM_2.5_ around the informal brick kilns in the Vhembe District from March 2021 to February 2022, using a network of well calibrated Dylos DC1700 PM monitors.
- To examine the compliance of the PM_2.5_ levels in the host communities of the brick kilns to the South African National Ambient Air Quality Standard (NAAQS).
- To determine the temporal variation of PM_2.5_ level.
- To investigate other potential sources of the PM_2.5_ in the study area.

## Methodology

### The study area

**Figure 1:**
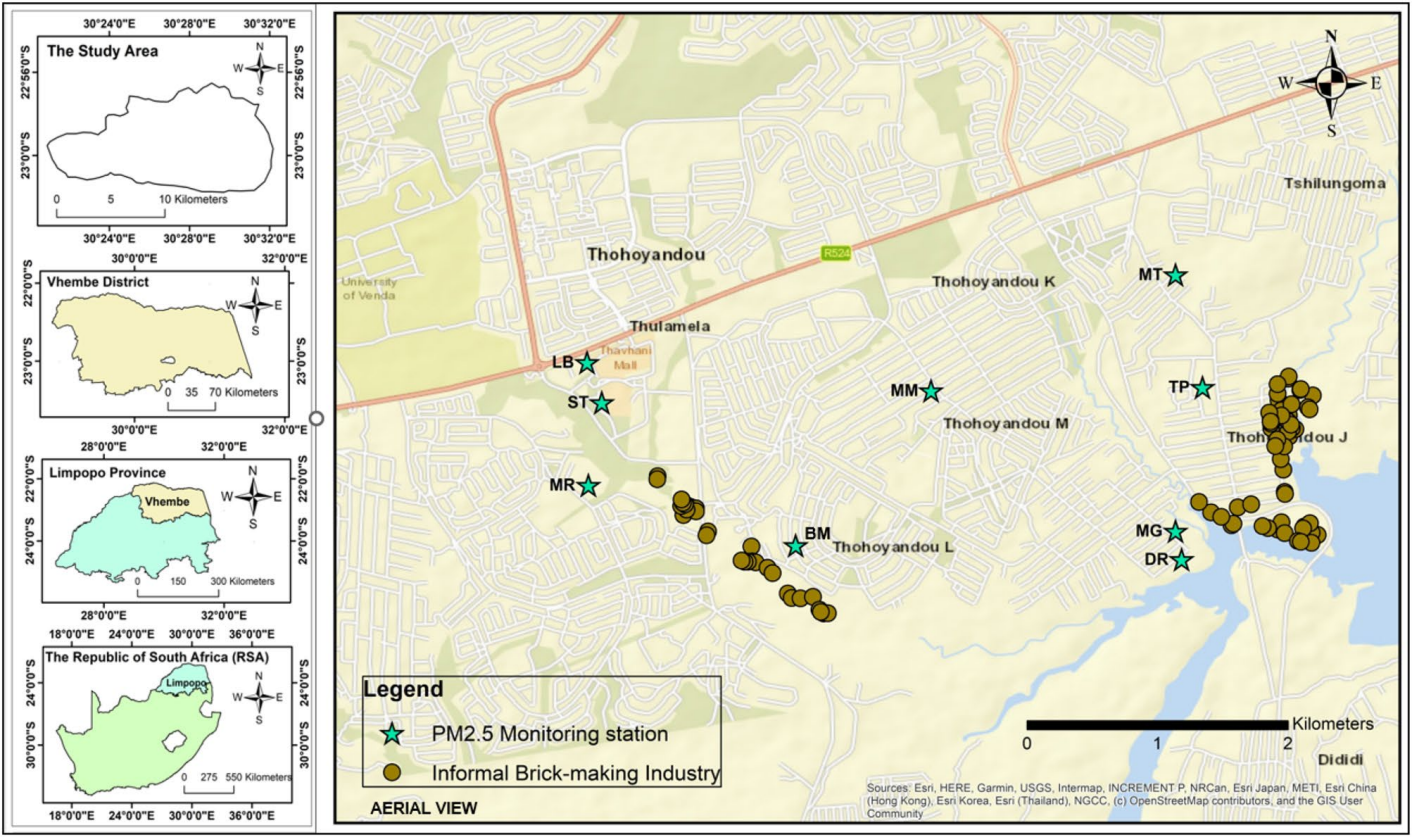
The study area map showing the geographical locations of the monitoring stations produced using ArcGIS 10.8.

The study area for this work is the Vhembe District in northern Limpopo province (22.7696°S, 29.9741°E), South Africa as shown in Fig. 1. Through the Kruger National Park, Vhembe District shares borders with Zimbabwe and Botswana in the north-west and Mozambique in the south-east. The area is separated from its bordering countries by the Limpopo River. The district is divided into four municipalities: Mahkado, Collins Chabane, Musina, and Thulamela. It has a vast amount of territory that spans 25,596 km^2^. Its population is estimated at 1.5 million people. The populace primarily relies on mining and agriculture for its subsistence^56^. Vhembe District receives most of its rainfall between October and March^57^. The district receives between 400 and 1100 mm of precipitation annually. The district also experiences the highest temperature during the wet season^57^. Vhembe District has many informal bricks making industries, employing the use of the clamp kiln for firing of bricks. The brickmaking industries are often in clusters, and they are mainly situated in Mannini and Tshilungoma villages. These areas of the district are desirable places for brick producers due to the presence of clay deposits and water bodies^51,58^.

### Site selection and instrument installation

Before the commencement of the study, Ethical clearance (SES/19/ERM/08/1309) was obtained from the University of Venda Research and Ethics Committee. Nine locations within 2 km radius from the clusters of informal brick kilns were selected for installation of PM_2.5_ monitoring equipment. The monitoring sites were selected based on approval from the municipality, ward counsellors, traditional rulers, and other constituted authorities/landowners. Another important factor considered for selecting the monitoring sites was the security of the monitoring instruments to be installed. The location of the PM_2.5_ monitoring stations were selected to span a range of land use characteristics, including governmental facilities/organizations, academic environments, recreation centre, site near local and arterial roads, and residential areas (Table 1).

**Table 1:** The selected sites and their characteristics.

Site	Latitude	Longitude	Land use	Distance from brick kilns (Km)	Potential PM_2.5_ emission sources
MR	− 22.992155	30.460613	Student Lodge	1.0	Paved and unpaved roads, bush burning, brick firing
LB	− 22.983674	30.460521	Municipality Library	1.6	Vehicular emission
ST	− 22.986445	30.461542	Municipality Stadium	1.3	Paved and unpaved roads, brick firing
BM	− 22.996322	30.474917	Residential building	0.5	Brick firing, biomass burning, unpaved road
MM	− 22.985625	30.484258	Creech/ Primary school (100 m away from T- junction)	1.9	Vehicles, paved and unpaved roads
DR	− 22.997290	30.501529	Recreation centre/Lodge	0.7	Brick firing, bush burning, unpaved road
MG	− 22.995323	30.501120	Residential building	0.6	Brick firing, bush burning, unpaved road, garbage combustion
TP	− 22.985395	30.502983	Primary School	0.8	Bush burning, brick firing, unpaved road
MT	− 22.977612	30.501116	Residential building	1.7	Biomass burning for cooking, brick firing

**Figure 2:**
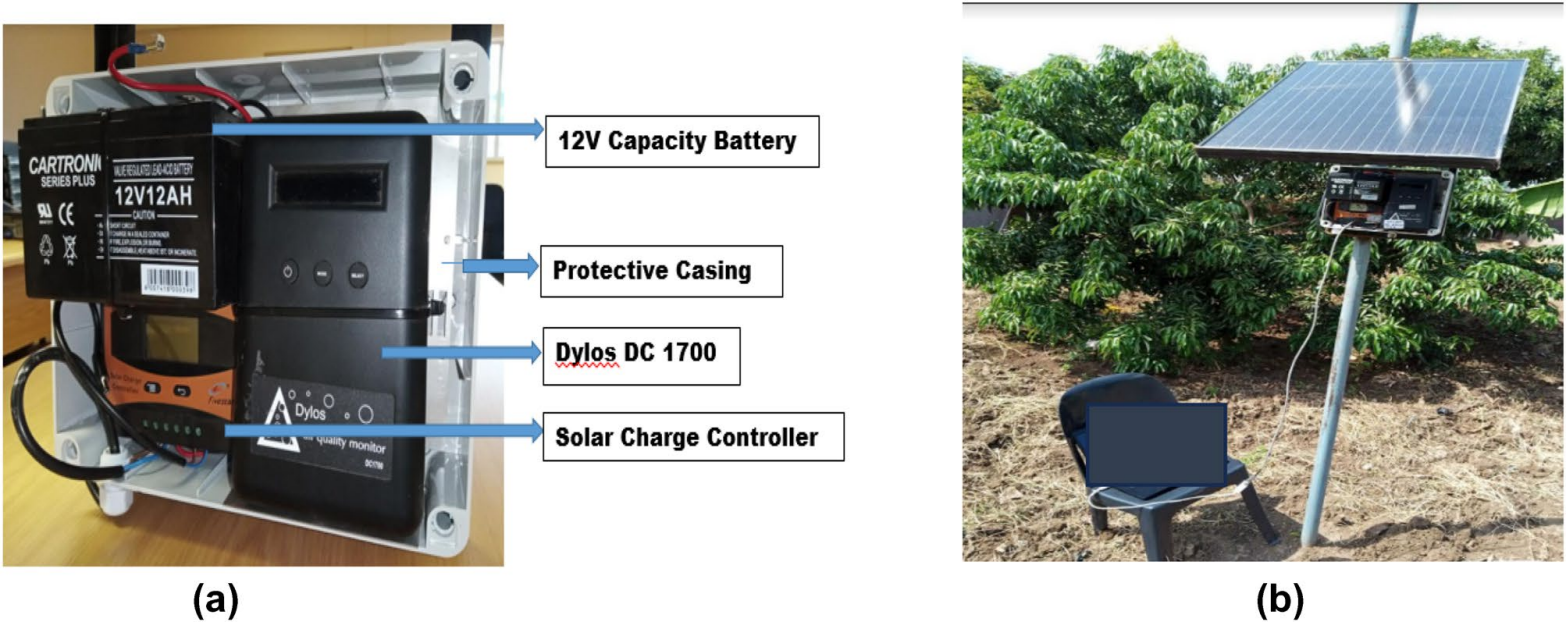
The monitoring equipment (**a**) Modified air monitoring device. (**b**) Installed air monitoring device connected to a laptop.

Prior to Dylos DC1700 (Dylos Corporation, Riverside, CA) installation, the Dylos units were modified by the technical crew of the Climatology Research Group at the North-West University, Potchefstroom, South Africa. A solar panel, solar controller, higher voltage battery (12V–12A), and a waterproof housing was added to each of the Dylos devices to enhance their performance in the outdoor environment (Fig. 2a). One of the modified Dylos units was then collocated with a GRIMM Environmental Dust Monitor 180 at the North-West University for a period of 5 months. A correction factor was generated from the collocation exercise of the Dylos device with the GRIMM instrument, and this factor was used to estimate the Particle Mass Concentration (PMC) in µgm^−3^ in the study.

For the installation of the monitoring devices at the selected points, EPA Probe and Monitoring Path Siting Criteria for Ambient Air Quality Monitoring was followed as practicable as possible. The instruments were installed at 1.5 m above ground level (Fig. 2b), about 1 m from wall/fence and 10 m from the drip line of trees^59^. Height of 1.5 m above ground level is assumed to be the statistical average breathing height of pedestrians^60–63^. One-year continuous monitoring of PM_2.5_ was conducted from March 2021 to February 2022. For the study, hourly meteorological measurements for the study period (March 2021–February 2022) were obtained from the South African Weather Stations (SAWS) situated within the study area (Latitude − 22.9160 and Longitude 30.4050). The meteorological parameters used for the study are the wind direction and wind speed.

### Dylos DC1700 instrumentation

The Dylos device is programmed to collect air quality data from the sampled air stream every minute. Data were collected and stored in the memory throughout the stipulated monitoring duration. Due to the short battery capacity of Dylos DC1700, the internal battery of the device was complemented by a solar system with 12 voltage battery. Readings on the Dylos device are displayed in 2 size bins; one counting all particles greater than 0.5 µm (the detection limit) and the other counting only coarse particles above 2.5 µm^34^. Particle number count (PNC) between 0.5 and 2.5 µm is calculated from the difference between the PNC of the large (> 2.5 µm) and small (> 0.5 µm) particles^22,34^. Measurement with this device is expressed in particle count per 0.01 cubic foot (0.283 L) of air^64^. Given that there is basically no epidemiological proof connecting PNC with health impacts, and to communicate the implications of exposure to poor air quality to society^64,65^, it is essential to report the measurement from the Dylos device in the corresponding mass concentration. The corresponding mass concentration could be estimated using the correction factor generated during the collocation experiment^34,66^. The device is provided with exclusive programming Dylos Logger (V.1.60) to empower the logged information from the instrument to be downloaded^64^. The measured data are automatically stored in internal memory and downloaded through a serial port to a computer using a 9-pin serial/Universal Serial Bus (USB) cable^64,67^. Due to the short memory capacity of the device, stored data were downloaded manually at least once a week.

### Quality control and data analysis

To ensure data quality, the air quality monitoring exercise was subjected to recognized procedures so that the resultant data are representative and comparable. The method described by Aniyikaiye et al.^66^ was used. A quality control check was carried out on the collected data by removing the negative and zero readings. The minute-by-minute air quality data from the installed Dylos devices were collated and converted to the PMC using the correction factor generated from the collocation exercise. The correction factor was generated using Eqs. (1) and (2).

1 $${PNC}_{PM2.5}={PNC}_{small}-{PNC}_{large}$$

2 $$PMC=0.0059\left({PNC}_{PM2.5}\right)+12.977$$

where PNC_PM2.5_ is the particle number between 0.5 and 2.5 µm. PNC_small_ is the particle number count > 0.5 µm particles generated by Dylos DC1700. PNC_large_ is the particle number count > 2.5 µm particles generated by Dylos DC1700. PMC is the calculated Particle mass concentration in µgm^−3^.

Generally, a dataset comprising of at least 80% of data capture is required for quality assurance and to qualify for data manipulation and summary^68,69^. However, this was lowered to accommodate the modest functionality of the low-cost instrument. For collation of the daily and monthly data, 70% data completeness was used. In order words, days and months with less than 17 hours and 21 days available data, respectively, were discarded and not included in the data analysis process. The hourly PM_2.5_ and meteorological data were synchronised and processed to prepare datasets with different time scales (diurnal, daily, monthly, and seasonal time scales). Excel 2016, R, and openair R statistical packages were used to analyse the collected data. The relationship and trends of PM_2.5_ concentrations and meteorological parameters were examined using the following functions: ggplot2, timeVariation, and polarPlot, and the results were discussed in terms of the overall contribution of local sources (Brickmaking industries inclusive) to the atmospheric PM_2.5_ levels at geographically different locations. The temporal distributions of PM_2.5_ across the PM_2.5_ monitoring stations, and the compliance levels of PM_2.5_ concentrations from the monitoring stations to the South African NAAQS for PM_2.5_ were determined. Also, the directional information on potential PM_2.5_ sources were assessed.

### Local/ regional transport analysis of PM_2.5_

Meteorology greatly influences the level of pollutants in ambient air. Among the numerous meteorological factors, wind speed and direction play significant role in controlling the pollutants concentrations in the atmosphere. Bivariate polar plots are often employed in illustrating the variability of pollutant concentration with wind speed and wind direction in polar coordinates^70^. In a bivariate polar plot, wind speed is represented as the distance from the origin and wind direction as the angle from the origin. The varied colours in the polar plot represent the average concentrations of the pollutant variable^71,72^. For the study, the local and regional transport analysis of PM_2.5_ was determined using bivariate polar plots (BPP). The PM_2.5_ hourly data obtained from the monitoring stations as well as the corresponding hourly meteorological data for the period of study were used for the BPP.

## Result and Discussion

### Collocation of GRIMM EDM 180 Against Dylos DC1700

**Figure 3:**
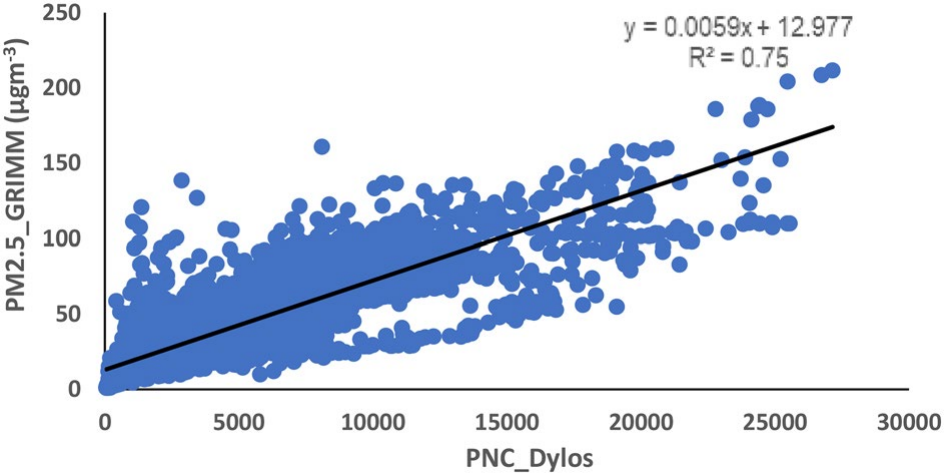
Relationship between the GRIMM EDM 180 and Dylos DC1700 readings during the collocation exercise.

A coefficient of determination, R^2^ = 0.75 was attained between the 10 min readings and the corresponding 10-min averaged PNC generated by the GRIMM reference instrument and the Dylos device, respectively, during the 5-months collocation exercise held at the North-West university monitoring station in 2021 (Fig. 3). In addition, high reproducibility levels with R^2^ values between 0.99 and 1 were attained between the 1-h PM_2.5_ average concentrations generated by all Dylos sensors used during a 5-days calibration exercise held at the North-West University in 2020^73^. High R^2^ value of pollutant concentrations among network of similar instruments are indication of consistency and strong similarity in the readings from the instruments^74^. With reference to the Dylos PM sensors, the high R^2^ value implies that the Dylos units can generate similar set of readings when subjected to the same environmental condition. For this study, the linear equation generated from the collocation exercise between the Grimm EDM 180 and Dylos DC1700 is referred to as the correction factor. The correction factor was determined using Eqs. (1) and (2) above. The correction factor was used for the conversion of the PNC from the PM_2.5_ monitoring stations to PMC (particle mass concentration) in microgram per cubic meter.

### Data

The summary of data availability for all the PM_2.5_ monitoring stations within the period of study are presented in Table 2. 70% data completeness is the threshold, data availability with percentages less than 70 are emboldened, while the empty spaces represent results that were not available due to theft of solar panel. For data analysis, the months with less than 70% data were disregarded and discarded. It is worthy to note that no data was available in October 2021 in all the monitoring stations, which was as a result of high rainfall and low sunlight levels. In addition, the low resolution of data collected between August and November was due to battery failure, which was latter replaced resulting in a much-improved dataset in the subsequent months. Based on findings from the study, Dylos performed best when there was no rain, and the batteries are fully charge. Other reasons for gaps in data for the sampling duration (March 2021–February 2022) include; Dylos turning off automatically, inadequate power supply from the solar panel to the equipment due to insufficient sunlight especially during winter (June, July and August) and spring (September, October and November (SON)), dead external battery, malfunctioning instrument, where the instrument was removed for repair, zero readings from the instrument and theft of the instrument.

**Table 2:** Data completeness for all the PM_2.5_ monitoring station from March 2021–February 2022.

	2021										2022	
Site	Mar	Apr	May	Jun	Jul	Aug	Sep	Oct	Nov	Dec	Jan	Feb
LB	91.8	100.0	99.9	100.0	94.2	91.4	87.5	**21.2**	**−**	**−**	**−**	**−**
ST	92.2	100.0	95.2	**69.2**	**12.5**	**7.0**	**3.3**	**2.4**	**39.4**	99.3	100.0	99.9
MR	85.5	85.3	**20.8**	**7.6**	**50.4**	**46.6**	**15.3**	**10.3**	90.6	99.6	100.0	99.9
MM	99.7	96.1	80.4	100.0	89.0	75.0	79.7	**15.3**	**39.7**	100.0	**62.4**	83.8
BM	85.6	100.0	94.5	73.6	73.0	**7.1**	**1.1**	**17.9**	**24.2**	**44.9**	**68.0**	**57.9**
DR	99.5	100.0	71.5	100.0	84.0	95.7	95.4	**65.2**	88.5	73.7	100.0	100.0
MG	99.3	100.0	99.9	100.0	89.1	**60.5**	**24.0**	**9.5**	**38.6**	99.9	100.0	100.0
TP	99.9	100.0	83.1	**37.6**	**34.8**	**37.9**	**19.3**	**4.8**	89.6	99.6	100.0	100.0
MT	95.7	100.0	**67.1**	**62.8**	**42.5**	**18.4**	**7.1**	**42.1**	**48.9**	71.9	93.4	**53.9**

Data availability with percentages less than 70% are emboldened.

Based on the prevailing wind direction, the monitoring exercise was conducted at “upwind” and “downwind” locations to the clusters of informal brick kilns. The upwind/downwind monitoring was intended to provide a measure of the contribution of the brick kiln operations to local PM_2.5_ concentrations. Therefore, two and seven monitoring sites situated upwind and downwind the brick kilns, respectively, were used to assess the PM_2.5_ levels of the host communities of the informal brick kilns.

For easy analysis, the monitoring stations were subdivided into two groups namely:

The group upwind the brick making industries (DR and MG).

The group downwind the brick making industries (BM, LB, MM, MR, MT, ST, TP).

Although data quality control considerably decreased the amount of data available for analysis of most of the locations, locations having at least one valid month in a season was assumed to be representative of that season. For monitoring stations situated downwind the informal brick kilns, PM_2.5_ concentrations were recorded at all the stations in autumn (March, April and May (MAM)). Conversely, there was low level (< 70%) of data capture at monitoring sites TP, ST, MT and MR in winter, BM, MT and ST in spring as well as BM and LB in summer (December, January and February (DJF)) due to equipment malfunction and theft.

### Daily variability pattern of PM_2.5_ in Vhembe District

The daily mean mass concentration of PM_2.5_ ranges from 18.6 to 28.4, and 23.5 to 31.7 μgm^−3^ for the groups of monitoring stations situated upwind and downwind the brick kilns, respectively (Table 3). The rows of data italicised in Table 3 are the seasons which were not represented in overall seasonal plots for the monitoring stations due to limited available monthly data because the season do not have at least 1 month with 70% data completeness. The daily average ambient mass concentrations of PM_2.5_ at all the monitoring station reached their maximum during winter (June, July and August (JJA)). The concentrations drop to their minimum during summer for majority of the stations, and in spring at stations ST and LB. Maximal concentrations of PM_2.5_ were observed across all the monitoring stations in winter due to the maximal operation of the brick kilns, domestic biomass combustion for cooking and space heating and stable atmospheric condition (Table 4). On the other hand, the reduced levels of PM_2.5_ experienced during summer and spring could be due to rainfall. In addition, high variability in PM_2.5_ concentration were observed in majority of the stations in winter. The relatively high variation in PM_2.5_ in winter compared to other seasons could be partly attributed to the occurrence of more severe PM_2.5_ pollution events coupled with the regular pollution events common in other seasons. The daily mean mass concentrations of PM_2.5_ were compared with the daily NAAQS of 40 μgm^−3^ for PM_2.5_. Based on the available data, maximum number of daily exceedances to the daily NAAQS limit for PM_2.5_ occurred in all the monitoring stations during winter while PM_2.5_ levels fell below the NAAQS for PM_2.5_ throughout the study area in summer.

**Table 3:** Descriptive statistics for the daily averaged ambient PM_2.5_ mass concentrations in µgm-3 at nine monitoring stations in Vhembe District, for the study period March 2021 to February 2022, sub-categorised by season.

Group	Site	Period	N	Data complete-ness (%)	Mean (µgm^−3^)	SD (µgm^−3^)	Median (µgm^−3^)	Range (µgm^−3^)	99^th^ percentile (µgm^−3^)	No of Daily Exceedances
Upwind	DR	Autumn (MAM)	82	89.1	28.6	7.1	27.2	15.3—53.9	52.8	5
		Winter (JJA)	84	91.3	36.9	7.6	38.0	16.4—57.5	53.4	33
		Spring (SON)	75	82.4	25.9	6.4	24.3	14.2—39.6	39.1	0
		Summer (DJF)	82	91.1	20.9	3.3	20.7	14.6—28.0	27.8	0
		Annual	323	88.5	28.2	8.6	26.1	14.2—57.5	51.9	38
	MG	Autumn (MAM)	91	98.9	31.2	7.4	30.6	17.2—55.4	53.3	7
		Winter (JJA)	70	76.1	37.8	7.6	39.0	21.7—55.7	53.8	29
		*Spring (SON)*	*13*	*14.3*	*20.1*	*5.8*	*18.0*	*14.0—35.0*	*34.2*	*0*
		Summer (DJF)	89	98.9	19.4	2.7	18.9	14.3—27.9	24.9	0
		Annual	263	72.1	28.4	9.8	26.3	14.0—55.7	53.0	36
Down-wind	MR	Autumn (MAM)	55	59.8	26.9	4.9	25.8	16.9—37.5	37.3	0
		*Winter (JJA)*	*25*	*27.2*	*35.2*	*6.9*	*35.5*	*18.3—48.7*	*48.1*	*7*
		Spring (SON)	32	35.2	25.8	6.4	24.8	15.0—51.3	47.2	1
		Summer (DJF)	90	100.0	21.1	2.9	20.6	14.9—28.8	27.6	0
		Annual	202	55.3	25.2	6.6	23.8	14.9—51.3	46.0	8
	ST	Autumn (MAM)	88	95.7	27.4	5.6	27.1	15.1—45.0	44.4	4
		*Winter (JJA)*	*20*	*21.7*	*32.1*	*4.7*	*33.5*	*21.4—40.7*	*40.2*	*1*
		*Spring (SON)*	*11*	*12.1*	*19.7*	*3.6*	*19.4*	*14.6—25.8*	*25.7*	*0*
		Summer (DJF)	90	100.0	21.1	2.9	20.9	14.8—27.5	27.1	0
		Annual	209	57.3	24.8	5.9	23.6	14.6 – 45.0	43.2	5
	LB	Autumn (MAM)	89	96.7	30.1	6.5	29.2	19.2—52.7	49.0	7
		Winter (JJA)	87	94.6	35.1	6.9	33.9	20.4—53.6	52.4	22
		Spring (SON)	31	34.1	29.9	5.1	29.3	18.0—42.2	41.3	1
		*Summer (DJF)*	*–*	*–*	*–*	*–*	*–*	*–*	*–*	*–*
		Annual	207	56.7	32.2	6.9	31.2	18.0—53.6	52.0	30
	BM	Autumn (MAM)	85	92.4	32.8	7.5	32.3	20.3—57.0	54.5	11
		Winter (JJA)	41	44.6	41.5	6.9	40.5	30.7—60.7	58.9	23
		*Spring (SON)*	*9*	*9.9*	*27.7*	*6.6*	*26.1*	*19.4—37.8*	*37.8*	*0*
		*Summer (DJF)*	*45*	*50.0*	*21.7*	*2.9*	*21.3*	*15.0—27.6*	*27.6*	*0*
		Annual	180	49.3	31.7	9.5	30.8	15.0—60.7	56.3	34
	MM	Autumn (MAM)	83	90.2	31.3	6.1	30.9	15.9—46.3	44.3	8
		Winter (JJA)	79	85.9	37.3	6.9	36.9	22.9—56.9	56.3	24
		Spring (SON)	37	40.7	28.1	7.3	27.6	15.6—47.4	44.5	1
		Summer (DJF)	73	81.1	22.5	3.3	22.4	15.8—29.8	29.3	0
		Annual	272	74.5	30.2	8.2	29.1	15.6—56.9	50.5	33
	TP	Autumn (MAM)	86	93.5	27.0	5.1	26.7	15.4—39.7	39.5	0
		*Winter (JJA)*	*24*	*26.1*	*30.8*	*5.3*	*29.6*	*17.3—40.8*	*40.1*	*1*
		Spring (SON)	30	33.0	21.2	3.4	21.2	13.9—30.2	29.0	0
		Summer (DJF)	90	100.0	19.0	2.4	18.8	14.5—25.4	24.7	0
		Annual	230	63.0	23.5	5.9	22.2	13.9—40.8	39.0	1
	MT	Autumn (MAM)	86	93.5	26.3	5.0	26.1	15.6—43.1	42.5	3
		*Winter (JJA)*	*38*	*41.3*	*28.1*	*7.6*	*28.6*	*13.0—47.2*	*45.6*	*2*
		*Spring (SON)*	*24*	*26.4*	*23.5*	*3.2*	*23.2*	*18.7—30.8*	*30.5*	*0*
		Summer (DJF)	62	68.9	20.4	2.8	20.1	16.0—27.3	27.1	0
		Annual	210	57.5	24.6	5.7	24.1	13.0 – 47.2	42.9	5

The rows of data italicised are the seasons.

**Table 4:** Variation in PM_2.5_ across the sampling months (mean ± sd) in microgram per cubic meter.

Season	Month	DR (µgm^−3^)	MG (µgm^−3^)	MR (µgm^−3^)	LB (µgm^−3^)	ST (µgm^−3^)	BM (µgm^−3^)	MM (µgm^−3^)	TP (µgm^−3^)	MT (µgm^−3^)
Autumn	Mar-21	23.9 ± 3.6	25.7 ± 4.3	23.6 ± 3.8	25.4 ± 3.5	22.6 ± 3.4	26.4 ± 4.3	27.0 ± 4.2	23.1 ± 3.5	22.9 ± 3.8
	Apr-21	28.5 ± 3.5	31.0 ± 4.2	29.4 ± 4.2	29.1 ± 3.9	27.7 ± 3.2	32.0 ± 4.6	32.2 ± 5.6	27.8 ± 3.5	26.9 ± 3.3
	May-21	35.6 ± 8.9	37.0 ± 8.2	–	35.1 ± 7.5	32.5 ± 6.3	39.9 ± 9.1	37.1 ± 7.5	32.0 ± 6.5	–
Winter	Jun-21	37.4 ± 6.7	38.4 ± 7.2	–	35.4 ± 7.9	–	42.6 ± 8.6	38.1 ± 8.4	–	–
	Jul-21	36.6 ± 7.8	36.4 ± 8.1	–	36.1 ± 9.9	–	40.6 ± 8.7	37.5 ± 6.6	–	–
	Aug-21	35.9 ± 9.1	–	–	35.0 ± 8.0	–	–	37.2 ± 9.5	–	–
Spring	Sep-21	30.1 ± 7.6	–	–	29.3 ± 6.1	–	–	31.7 ± 5.9	–	–
	Oct-21	–	–	–	–	–	–	–	–	–
	Nov-21	22.0 ± 3.6	–	24.9 ± 7.0	–	–	–	–	20.4 ± 3.3	–
Summer	Dec-21	21.1 ± 3.9	19.0 ± 2.7	21.8 ± 3.4	–	22.2 ± 3.2	–	22.6 ± 3.7	19.3 ± 2.9	20.2 ± 3.4
	Jan-22	20.0 ± 2.8	18.8 ± 2.5	19.8 ± 2.1	–	19.7 ± 2.1	–	–	18.3 ± 2.0	19.9 ± 2.2
	Feb-22	21.6 ± 3.3	20.3 ± 3.0	21.6 ± 2.7	–	21.6 ± 2.6	–	22.9 ± 3.5	19.5 ± 2.3	–

### Monthly/ seasonal characteristics of PM_2.5_

Considering the monthly variation in PM_2.5_ in both the upwind and downwind direction of the brick kilns, relatively higher level of PM_2.5_ were observed from late autumn (May) and during winter which are periods of expected high pollution. Studies have shown relatively high levels of ambient pollution during winter compared to pollution level in rainy season^75–77^. The abrupt increment of wintertime pollution especially PMs concentrations, is primarily ascribed to extensive use of fuel during winter for large-scale heating^31,32,68,78,79^. Previous findings by Aniyikaiye et al.^51^ (2021a) in the study area also indicated highest levels of the brickmaking industrial operations during winter. June and July are the key winter months in the study area. PM_2.5_ generating activities in this area such as brick firing, domestic biomass combustion for cooking and space heating are highest during autumn and winter. Maximum PM_2.5_ level was observed in June in majority of the stations with winter data except LB which occurred in July (Table 4).

**Figure 4:**
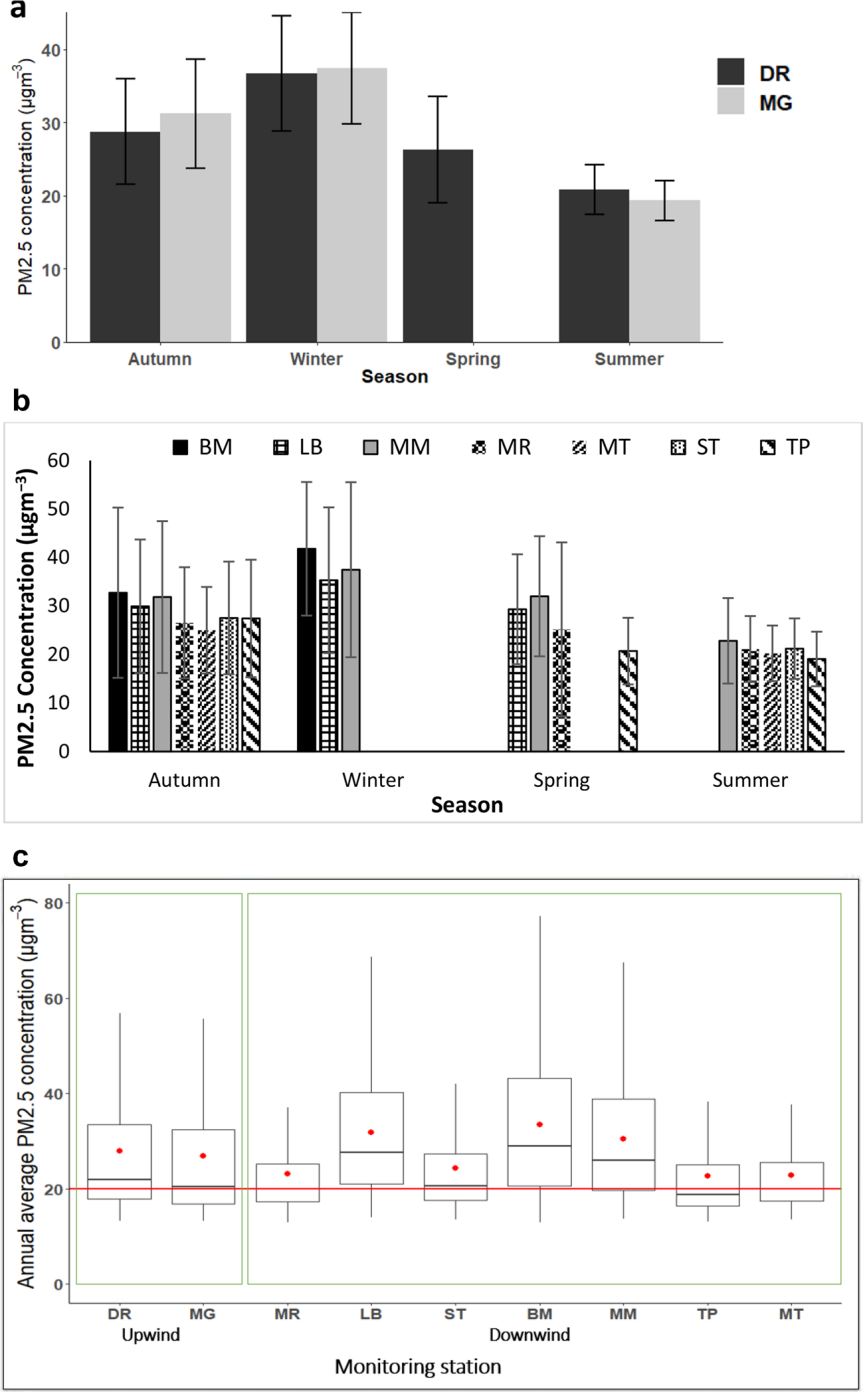
Temporal variation in PM_2.5_. (**a**) Seasonal variation in PM_2.5_ concentration of the monitoring stations in the upwind direction of the brickmaking industries and the standard deviations (bars). (**b**) Seasonal variation in PM_2.5_ concentration of the monitoring stations in the downwind direction of the brickmaking industries and the standard deviations (bars). (**c**) Annual ambient PM_2.5_ compliance status of monitoring stations close to the brick kilns in Vhembe District. The box-and-whisker plots show the minimum and maximum values (the lower and higher end of the whiskers); means (red dots); 25th, 50th, and 75th quartiles; and the annual NAAQS threshold of PM_2.5_ (solid red line).

Based on the available data, PM_2.5_ levels increased from summer < spring < autumn < winter at all the monitoring stations around the brick kilns. During the wet season (summer and spring), brickmaking activities are reduced in Vhembe District due to unconducive weather condition for brick production, and water lodging of the brickmaking areas^51^. Mazumdar et al.^80^ also claimed water lodging of quarried land as a major challenge on brick making industries during the rainy season at Palasbari Revenue Circle, India. The reduced brickmaking activity during the wet season could have contributed to the low level of PM_2.5_ recorded in summer and spring. Additionally, the occurrence of favourable meteorological conditions, such as increased temperature and wind speed as well as high precipitation levels during these seasons aid pollutant dispersion and reduction. According to Yuan et al.^81^, precipitation is one reason for low pollutant contamination in the wet season as the pollutants are washed out of the atmosphere. Figure 4a,b present the seasonal PM_2.5_ levels for the respective groups.

Comparing this study with similar studies in Africa, low to high levels of PM_2.5_ were reported (Table 5). There is disparity in air quality results between study conducted by Novela et al.^82^ and the current study despite the location of the study areas in the same district municipality. Unlike this study, which was conducted in a semi-industrialised setting, that of Novela et al.^82^ was in a control environment with limited PM_2.5_ emission sources. This study as well as other similar studies enlisted in Table 5 indicated reduced levels of PM_2.5_ during rainy season (spring and summer) and relatively higher PM_2.5_ concentrations during dry season (autumn and winter).

**Table 5:** Comparison of result from Vhembe District to that from other cities in Africa.

Country	Instrument	Duration/ Time	Number of monitoring points	Ambient mean PM_2.5_ Concentration (µg/m^3^)	Source
Kinshasa and Brazzaville, Republic of Congo	PurpleAir sensor	2018–2020	5	Rainy season—40	McFarlane et al.^83^
				Dry season – 60–70	
Yaoundé, Cameroon (landfill)	digital Aeroqual Dust Sentry	February 2016	11	Daily- 12.85–37.57	Feuyit et al.^84^
Abattoir, Nigeria	Aerosol Mass Monitor (831, U.S.A)	6am-2pm October–November	3	Daily- 18.75	Odekanle et al.^85^
Lagos, Nigeria	Airmetric minivol samplers PM2 5	2007–2008 (5 months)	8	26.68–272.85	Adeleke et al.^86^
Accra, Ghana	DustTrak Model 8520 monitors (TSI Inc.)	3 weeks	4	24h- 22.3–40.2 (Average- 27.4)	Arku et al.^87^
Zambia	Gravimetry, instrument not stated	July 2015–February 2016	2	2.39–24.93	Nkhama et al. ^88^
Bethlehem, South Africa	Grimm Dustcheck 1.108. portable dust monitor (light scattering)	July 2001		24h- 65	Worobiec et al. ^89^
Botsalano game reserve in North-West Province, South Africa	Tapered Element Oscillating Microbalance (TEOM) model 1400a (Rupprecht and Patashnick R&P, Co. Inc.)	July 2006–July 2007	Mobile	Annual median- 10.5	Laakso et al.^90^
Petrus Molefe Eco Park, Johannesburg, South Africa		October 2013, June–July 2014	Mobile	Spring- 18.1–61.2 Winter- 62.9–126	Valsamakis^91^
Thokoza Park, Johannesburg, South Africa				Spring- 18.5–38.4 Winter- 25.1–71.9	
University of Venda, Thohoyandou, South Africa	GilAir-5 personal air samplers (gravimetric instrument)	April 2017–April 2018	1	Autumn- 10.41 Winter- 9.83 Spring- 14.69 Summer- 8.64 Annual- 10.89	Novela et al.^82^
Host communities of the informal brick kilns in Vhembe District, South Africa	Dylos DC 1700	March 2021–February 2022	9	Autumn—24.8–32.9 Winter- 35.5–41.6 Spring- 20.4–31.7 Summer- 19.0–22.7 Annual- 22.6–36.2	This study

### Spatial distribution of PM_2.5_ in Vhembe District

The annual distribution of PM_2.5_ across the monitoring sites for the study period is presented in Fig. 4c. This study showed that ambient PM_2.5_ concentrations range of 22.6 (MT)–36.2 µgm^−3^ (BM). With respect to the compliance levels of the air quality from the monitoring stations to the annual NAAQS of PM_2.5,_ none of the annual average PM_2.5_ concentrations from all the monitoring sites fell below the annual NAAQS level of 20 µgm^−3^. DR and MG monitoring stations situated upwind the brick kilns are expected to have the least levels of PM_2.5_, however, annual average concentration of PM_2.5_ recorded in these monitoring stations were relatively higher than the annual averages of some monitoring sites (MR, ST, TP, and MT) situated downwind the brick kilns. The relatively higher PM_2.5_ concentrations recorded in MG and DR stations could possibly be attributed to the presence of other prominent sources of PM_2.5_ (other than the brickmaking industries) in the upwind direction of MG and DR monitoring stations.

Result from the study also showed that median values of majority of the monitoring stations namely; MG, MR, ST, TP, and MT were lower than or approximately equal to the annual threshold limit of 20 µgm^−3^. This implies that about 50% of the PM_2.5_ levels reported at the listed stations fell below the annual NAAQS threshold limit for PM_2.5_ throughout the monitoring period. On the other hand, only about 25% of the collected PM_2.5_ data were below the PM_2.5_ annual threshold limit at stations LB, BM and MM. This indicates that high PM_2.5_ levels were present in the areas surrounding these three monitoring stations virtually throughout the study period, exposing residents to health risks associated with PM_2.5_.

Similar studies on ambient air quality assessment around brick sites across the world showed moderate to high levels of PM_2.5_ at locations close to the brick kilns, which in most cases exceeded the NAAQS permissible limit of PM_2.5_ of the respective countries. Saha et al.^92^ in their study involving ambient PM_2.5_ monitoring around 12 selected brick kiln clusters in Rajashahi and Gazipur districts, between January to April, 2016 and 2017, obtained mean PM_2.5_ concentration range of 25.7–2298 µgm^−3^ across the selected sites. Similarly, high PM_2.5_ concentration range between 190 and 2305 µgm^−3^ was reported by Subhanullah et al.^93^ in a 2-days ambient monitoring of PM_2.5_ around brick kilns in Northern Pakistan. PM_2.5_ assessment studies around the brick kilns in Asia were not conducted within a year cycle, but only considered the active periods and operational hours of the brickmaking industries, hence, higher level of PM_2.5_ due to brickmaking were reported^49,92,93^. In addition, majority of the brick kiln used in those studies are Fixed Chimney Brick Kilns which are associated with relatively high release of PM_2.5_ compared to the South African clamp kiln^49,50^.

### Diurnal variability of PM_2.5_

**Figure 5:**
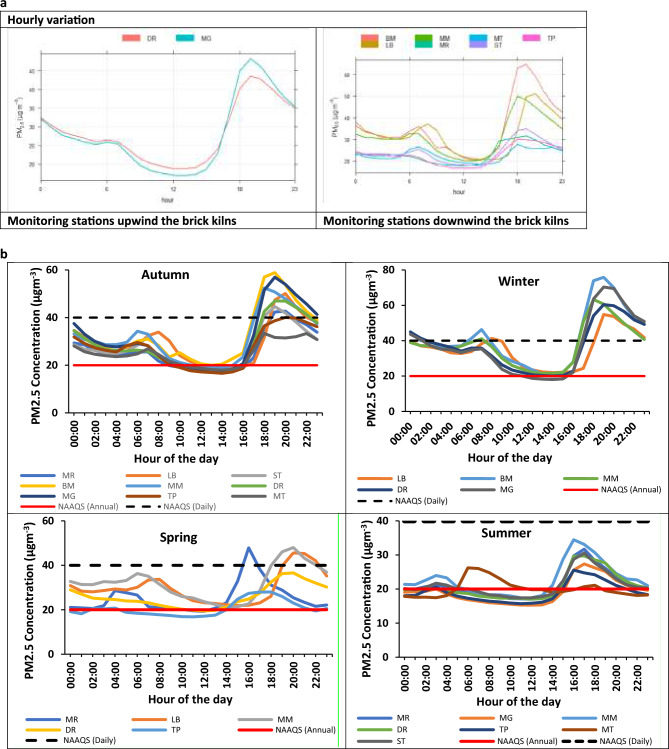
The diurnal variation of PM_2.5_ across the monitoring stations (**a**) Annual variability (**b**) Seasonal variability.

PM_2.5_ concentrations from the monitoring stations situated in the two groups showed similar diurnal trends (Fig. 5a). Two PM_2.5_ peaks were observed; in the morning (6h00–9h00), and evening (18h00–21h00) throughout the study period. The observed morning and evening PM_2.5_ peaks coincide with periods of high traffic congestion, biomass combustion for domestic and industrial purposes such as brick firing. Furthermore, the stable atmospheric condition in the evening (18h00) over the early hours of the day (6h00) could also have aggravated the extent of pollution around the brick kilns in Vhembe District. Similarity in the PM_2.5_ patterns generated by the upwind and downwind groups indicates the possible release of PM_2.5_ from similar sources. According to Vhembe District Municipality^94^ (2020/21) report, fugitive emissions from the brickmaking industries especially the clamp kiln is a major source of PM_2.5_ in Vhembe District, during winter. Other primary sources of PM_2.5_ emissions include the commercial and small-scale industries, emissions from vehicles, domestic fuel combustion, dust entrainment, biomass combustion, waste disposal and wildfire^94^.

The higher peak observed in the evening shows possible increased frequency of activities from PM_2.5_ sources at night. From the survey and interviews conducted with the brick kiln workers, brick firing was claimed to be carried out by majority of the brickmakers mostly in the evening when the ambient temperature is low, and occasionally in the morning^51^. Regarding the PM_2.5_ levels in monitoring stations in the upwind and downwind direction, concentrations of PM_2.5_ at the downwind direction for some of the stations were lower than monitoring station (DR and MG) situated in the upwind direction. This gives an indication of presence of more prominent PM_2.5_ source(s) upwind the monitoring stations DR and MG which could be responsible for the relatively higher PM_2.5_ concentrations.

### Diurnal variability of PM_2.5_ across the seasons

To gain a better understanding on the PM_2.5_ trends, their temporal variation across the communities of the brick kilns, the compliance levels as well as the potential sources, it would be needful to sub-categorise the diurnal plot based on seasons. The seasonal diurnal variabilities of PM_2.5_ with respect to their compliance levels to both the daily and annual NAAQS threshold of 20 and 40 µgm^−3^, respectively, are presented in Fig. 5b. Generally, two PM_2.5_ concentration peaks (morning and evening peaks) were observed across all seasons at all the monitoring stations with the evening peaks being more prominent. It is noteworthy that the morning peaks of PM_2.5_ at all the monitoring stations fell below the daily NAAQS all through the study period, except for few (three) monitoring stations during winter.

Based on the compliance levels of PM_2.5_ at the monitoring stations during autumn (March–May 2021), PM_2.5_ concentrations at all the monitoring stations fell below the annual and daily NAAQS thresholds between 13h00 and 15h00. Additionally, all the monitoring stations were in compliance with the daily NAAQS for PM_2.5_ between 0h00 and 17h00, while the evening peak in majority of the monitoring stations was non-compliant with both the annual and daily NAAQS threshold limits. In winter (June–August 2021), the highest level of diurnal PM_2.5_ concentrations were recorded at all the monitoring stations. In addition, the PM_2.5_ concentrations at all monitoring stations exceeded the annual NAAQS during the day except for MG, which fell below it between 12h00 and 15h30. The evening PM_2.5_ peaks (17h00–0h00) observed at all the monitoring stations exceeded the daily NAAQS limit.

During spring (September–November 2021), the intensities of some of the observed prominent sources of PM_2.5_ in the study area dropped drastically. For instance, the industrial operation of the brick kilns was greatly reduced in spring due to unfavourable weather conditions. Also, the combustion of biomass for space heating purpose is brought to a halt. Unlike the other seasons where the morning and evening peaks for all the monitoring stations occurred about the same period, varying PM_2.5_ peaks were observed at all the stations during the morning and the evening, with the variation in the evening peaks being more noticeable in spring. The drastic reduction in some of the prominent PM_2.5_ sources such as brickmaking, biomass combustion for space heating, might be responsible for varying PM_2.5_ peaks observed across the monitoring stations. Similar evening peaks (15h00–18h00) and (19h00–21h00) were observed at monitoring stations (MR and TP) and (MM, DR and LB), respectively. MR and TP are educational parastatals, high levels of PM_2.5_ in these monitoring stations correlates with peak periods of movement of vehicle conveying the student and staff members from school. On the other hand, MM and LB are situated close to T-junctions linked to network of roads. Maximal level of PM_2.5_ between 19h00 and 21h00 signifies peak period of vehicular congestion. DR is a lodge, peak PM_2.5_ concentration between 19h00 and 21h00 could be possibly from PM_2.5_ emission from vehicles bringing in the clients as this period corresponds with the arrival time of most of the customers.

During summer, generally low levels of below NAAQS daily threshold limit of 40 µgm^−3^ were reported in all the monitoring stations. However, from 6h00 to 15h00, majority of the monitoring stations fell below the annual NAAQS threshold limit. The NAAQS annual limit for PM_2.5_ was exceeded at all the monitoring stations only during the morning and evening peaks of PM_2.5_ in summer. Diurnal variability in similar studies have shown one/ two PM_2.5_ peaks, coinciding with peak periods of PM_2.5_ generating activities^95–101^. The summary of PM_2.5_ diurnal pattern in similar studies are presented in Table 6.

**Table 6:** Comparison of PM_2.5_ diurnal variability pattern in Vhembe District with similar studies.

City/Country/ Region	Monitoring Environment Type	Number of Peaks	Time resolution	Morning Peak	Evening Peak	Reference
Dhaka	Urban/ Regional	1	Annual	7:00–9:00	–	Dobson et al.^95^
Eastern Africa		2	Annual	7:00–9:00	22:00–0:00	
Eastern Asia		2	Annual	10:00–12:00	0:00–2:00	
London		2	Annual	6:00–8:00	20:00–21:00	
New York		2	Annual	6:00–8:00	18:00–20:00	
Paris		2	Annual	8:00–9:00	21:00–23:00	
South America		2	Annual	8:00–10:00	22:00–0:00	
Southeastern Asia		2	Annual	7:00–9:00	2:00–4:00	
Southern Asia		2	Annual	8:00–10:00	23:00–0:00	
Southern Europe		2	Annual	10:00–11:00	20:00–21:00	
China	Urban	2	Annual	10:00–11:00	22:00–23:00	Xu et al.^96^
Xian, China	Urban	1	Winter	10:00–14:00	–	Zhang et al.^97^
		1	Summer	4:00- 8:00	–	
China (Hefei/ Shanghai/ Wuhan/ Nanjing/ Hangzhou)	Urban	2	Winter	10:00–11:00	21:00–22:00	Dai et al.^98^
		2	Autumn/summer/ spring	8:00–9:00	22:00–23:00	
Tianjin, China	Urban	2	Summer	6:00–8:00	18:00–19:00	Chen et al.^99^
		1	Annual/ Spring/ Autumn/ Winter	8:00–10:00	–	
Ho Chi Minh City, Vietnam	Urban	1	Annual/ Dry/ Rainy	10:00–12:00	–	Hien et al.^100^
Beijing	Rural	2	Spring	7:00 – 9:00	19:00–21:00	Fu et al.^101^
		2	Summer	6:00–8:00	18:00–20:00	
		2	Autumn	7:00–9:00	18:00–20:00	
		2	Winter	8:00–10:00	21:00–22:00	
Vhembe District, South Africa	Rural	2	Annual	6:00–9:00	18:00–21:00	This study
		2	Autumn	5:00–9:00	19:00–21:00	
		2	Winter	6:00–9:00	18:00–21:00	
		2	Summer	2:00–4:00	17:00–19:00	
		2	Spring	5:00–9:00	17:00–22:00	

### Meteorological conditions and bivariate polar plot

**Figure 6:**
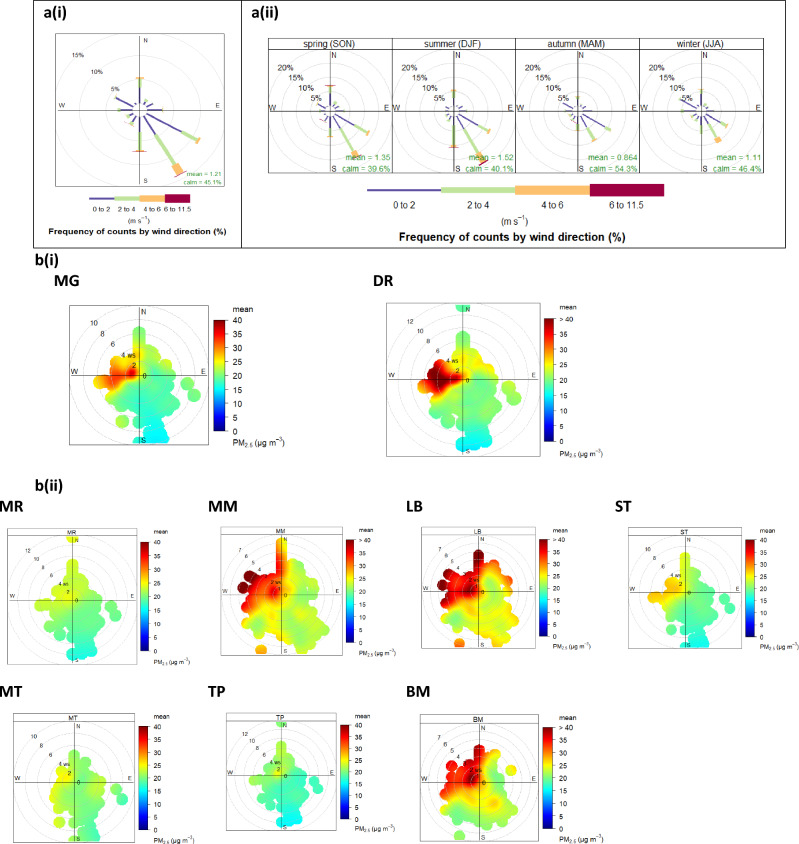
Meteorological conditions and bivariate polar plot. (**a**)(**i**) Annual wind rose of hourly data in Thulamela municipality. (**a**)(**ii**) Seasonal windrose of hourly data in Thulamela municipality (March 2021 to February 2022) indicating the prevailing wind direction, the mean windspeed and the percentage calmness. (**b**)(**i**) Polar plots of mean concentrations of PM_2.5_ for the monitoring period at the monitoring stations upwind the brickmaking industries. (**b**)(**ii**) Polar plots of mean concentrations of PM_2.5_ for the monitoring period at monitoring stations downwind the brickmaking industries.

Meteorology has a great influence on PM_2.5_ concentration, and its transportation from the source(s) to other locations in Vhembe District. Wind speed and direction provides directional information on the source(s) of PM_2.5_. Figure 6a(i) and (ii) illustrate the annual and seasonal wind roses for the study area during the period of study. Throughout the period of study, inflow of wind was predominantly from the south-east with the maximum wind speed from this direction in summer. High wind speed brings about the dispersion of PM_2.5_ away from the source(s), thus leading to the dilution and reduction in PM_2.5_ concentration within the given locality. On the other hand, reduced wind speed in a specified direction results in the accumulation of PM_2.5_ close to the source.

The influence of wind speed and direction on PM_2.5_ concentrations across the monitoring stations were assessed using the bivariate polar plots. The polar plot shows quite similar patterns for all the monitoring stations. Overall, high PM_2.5_ concentrations were observed at all the stations at the center, North, west and south-west, although at varying concentrations across the monitoring stations (Fig. 6b(i) & (ii)). At low to moderate wind speed of 1–5 ms^−1^, generally low PM_2.5_ levels ≤ 25 µgm^−3^ were observed at monitoring stations MR, MT, TP, moderate levels < 30, at station ST, and relatively high PM_2.5_ levels > 30 µgm^−3^ at stations MG, DR, MM, LB and BM in the aforementioned directions compared to the other regions around the monitoring stations. The high PM_2.5_ at the center, the northern, western and southwestern areas of the monitoring stations indicate the potential influence of the weak wind speed on PM_2.5_ concentration in these directions. Low wind speed facilitates the accumulation of PM_2.5_ released from local sources around the monitoring stations. The high concentration experienced close to the center at low wind speed ≤ 2 ms^−1^ at all the monitoring stations indicate PM_2.5_ levels from dominant local sources such as vehicular emission, and domestic biomass combustion. For all the stations, R534 arterial road situated northerly is a potential source while the high concentrations observed at south-western direction at wind speed > 6 ms^−1^ indicate possible release of PM_2.5_ from high rising stack of industries located in this direction.

For monitoring stations (MG and DR) situated upwind the brick kilns, similar trends in the polar plots were observed; highest PM_2.5_ concentrations > 35 µgm^−3^ were observed at the center and in the west direction at low to moderate wind speed of 1–5 ms^−1^. Potential PM_2.5_ sources at the center include domestic biomass and waste burning, bush burning, and vehicular source. Conversely, Thohoyandou township and Shayandima industrial estate situated in the west and southwest, respectively, are potential PM_2.5_ sources responsible for high PM_2.5_ levels at higher wind speed (> 6 ms^−1^) at these monitoring stations. According to the source apportionment study on PM_2.5_ conducted by Novela et al.^82^, urban emission and industrial sources account for 9.56% and 21.06%, respectively, of PM_2.5_ generated in Vhembe District.

For monitoring stations, downwind, the brick kiln, PM_2.5_ levels at monitoring stations MM, LB and BM showed similar trends. High concentrations > 30 µgm^−3^ were observed at the center, the northern and western regions of these monitoring stations. The high PM_2.5_ concentrations displayed at the center of monitoring stations MM and LB could possibly be from networks of road surrounding the monitoring stations. Recent study has reported about 10.31% of PM_2.5_ emissions originating from vehicular emissions within Vhembe District^82^. Conversely, the high PM_2.5_ concentration observed at station BM could possibly be attributed to domestic fuel combustion. Additionally, there were clusters of informal brick making industries which employ the use of wood-based clamp kiln for brick firing situated westly and south-westly the monitoring station BM. The clusters of informal brickmaking industries are potential sources responsible for the high PM_2.5_ in west and southwestern regions of station BM. Previous studies have revealed that wood combustion particles accounts for about 24.5% of particulate matters in Limpopo^102^. For monitoring stations LB and ST, construction sites situated at the southwestern region of these monitoring stations are potential sources, however, PM_2.5_ levels are mainly localised at monitoring stations MR, MT and TP.

### Limitation of the study

In this study, the regulatory standard required for quality assurance of PM_2.5_ data was lowered to accommodate the modest functionality of Dylos DC1700. For the collating PM_2.5_ data, 70% completion was used for the daily and monthly data collation instead of the approved data completion level of 80%. Whereas, for collation of the seasonal data, collated season must have at least one valid month. Invariably, all the monitoring stations were not represented throughout the seasons in the study period due to limited available data.

Other limitations include:

- Comparison of the monitoring sites was done based on unequal levels of data availability.
- Collocation of the Dylos device was only conducted at the North-west university.
- The collocation exercise was conducted for 5 months (June–October 2021) covering only two seasons (winter and spring) of the year.
- The PM_2.5_ data collected from the Dylos devices were not corrected to account for relative humidity (RH). In the future, it would be beneficial to investigate the impact of RH on the correction factor of Dylos DC1700.

## Conclusion

PM_2.5_ is of great challenge to scientists all over the world due to its short- and long-term effects on human health. Monitoring of PM_2.5_ creates awareness to the public about air quality around them, helping them to take necessary precautions. The conventional method involving the use of federal reference or federal equivalent methods is the most acceptable method. However, not all regions of the world can afford it. To enhance the understanding of the air quality around the less privileged regions of the world, the use of network of well calibrated low-cost sensors is a necessity. For the first time in Africa, a correction factor for PM_2.5_ readings from Dylos DC1700 particulate matter sensor was generated. Additionally, the long-term applicability of Dylos for continuous monitoring of outdoor PM_2.5_ was assessed. Findings from the study have shown that all the PM_2.5_ monitoring stations were non-compliant with the annual NAAQS threshold limit of 20 µgm^−3^ for PM_2.5_. However, about 50% of the PM_2.5_ concentrations at majority of the stations fell below the NAAQS annual threshold limit. Although, none of the monitoring stations exceeded the NAAQS thresholds in summer, highest PM_2.5_ concentrations and daily exceedances of the daily NAAQS limit were observed during winter across all the monitoring stations. The high PM_2.5_ level experienced especially in winter could be drastically reduced through interventions such as electrification, the provision of clean energy alternatives at subsidised rates, and public enlightenment. The polar plots of all the monitoring stations show similar trends with high PM_2.5_ concentrations majorly at the centre, north, west and south-west areas of the monitoring stations. PM_2.5_ concentrations were majorly localised at monitoring stations MR, MT and TP. The polar plot also showed maximal impact on PM_2.5_ concentration from brick kilns on monitoring station BM located downwind and ≤ 500 m from the brick kilns. For the other monitoring stations, influx of PM_2.5_ were observed at wind speed > 5 ms^−1^ at the southwest, signifying industries as the potential PM_2.5_ source. Other likely potential sources responsible for the high PM_2.5_ concentrations around the monitoring stations include construction site, major R534 road and Thohoyandou township. The reduced impact of PM_2.5_ emission from brickmaking industries at most of the monitoring stations displayed by the polar plot does not necessarily means PM_2.5_ emission from the brickmaking industries has no influence on the ambient PM_2.5_ level, however, there is high possibility of increased PM_2.5_ readings in areas very close to the brick kilns which were not included as monitoring stations. In conclusion, the application of Dylos DC1700 offers an excellent solution to bridging the data gap and establishing air pollution control policies for public health protection in areas with sparse air quality data.
